# Hereditary hemochromatosis: a neglected diagnosis in orthopedics: A series of 7 patients with ankle arthritis, and a review of the literature

**DOI:** 10.3109/17453670903035583

**Published:** 2009-06-01

**Authors:** Åke Carlsson

**Affiliations:** Department of Orthopaedics, Lund University, Malmö University HospitalMalmöSweden

## Introduction

Hereditary hemochromatosis (HH) is a not uncommon autosomal recessive and potentially life-threatening disease. The hemochromatosis gene was identified by Feder et al. in 1996. About 1 in 200 individuals is estimated to be homozygous for the most common mutation—C282Y/C282Y. In the classical form of the disease, cysteine is substituted by tyrosine at amino acid 282 in both alleles. The so-called compound heterozygoty is less common (representing about 10% of cases) but is also compatible with HH. Here, histidine is substituted by aspartic acid at amino acid 63 in one allele and cysteine by tyrosine at amino acid 282 in the other (C282Y/H63D).

Due to increased intestinal absorption, homozygotes develop iron overload but penetrance is very variable (McCune et al. 2006). Most orthopedic surgeons meet patients with undiagnosed HH on a yearly basis and an early diagnosis and treatment is important in order to avoid cirrhosis of the liver. Also, the risk of developing hepataocellular cancer is at least 20-fold higher than in their first-degree relatives (Elmberg et al. 2003). Fatigue and arthritis, unspecific and therefore often neglected, are common presentations whereas the classic bronze diabetes (darkened skin, diabetes, and cirrhosis) is the final stage of untreated HH that is very rarely seen (McDonell et al. 1999).

A survey of 2,851 patients with hemochromatosis showed that patients had consulted a physician after an average of 2 years of symptoms, and on average it took a further 10 years before the diagnosis was made (McDonell et al. 1999). This is unfortunate, since medical treatment—i.e. regular phlebotomy—is effective.

During the period 2001–2008, I replaced 93 ankles in 86 patients and 4 of these patients proved to have hemochromatosis. Here I have reviewed the orthopedic manifestations in 7 patients who had one or both ankles replaced during this period, 3 of whom had had their ankles replaced elsewhere.

### Cases

The original records from all orthopedics departments and units of gastroenterology involved were consulted and the author interviewed the patients at least twice.

All but 1 patient were below the age of 45 when they had their first joint symptoms, and there was usually a long patient’s and doctor’s delay before diagnosis and treatment. In 4 cases the hip, knee or ankle joint, and not the MP joints, was the first joint to become symptomatic. Only in one case (patient 5) did the joint symptoms start after the time that the diagnosis of HH had been made (Table 1).

**Table 1.** Demographic data, laboratory findings, and joints replaced in 7 patients with hereditary hemochromatosis

A	B	C	D	E	F	G	H	I	J	K	L	M	N	O	P
1	F	C282Y/C282Y	45	56	84%	4115		30	MCP **^c^** and finger joints	15	R+L 54	–	L 55 R 58	4	
2	M	C282Y/C282Y	53	61	100%	>1,900		40	knee	13	61	60	R 60 L 61	4	
3	F	C282Y/H63D	48 38	48	69%	241	b	25	hip	13	38	–	R 48	2	
4	F	C282Y/C282Y	62	71	Missing	1,426		52	MCP-joints **^c^**	10	–	R 66	L 71 R 72	3	
5	M	C282Y/C282Y	40	45	87%	3,200		45	knee and ankle	–	–	–	R 57	1 + HTO R+L**^d^**	2 knees 1 ankle
6	M	C282Y/C282Y	42**^a^**	42**^a^**	100%	2,456		20	multiple joint symptoms	22	–	–	R 49	1 1	1 ankle
7	M	C282Y/C282Y	44	44	99%	8,900		43	ankle	1	–	–	R 49	1	
Mean value			48	52	94% (n = 5)	3,666 (n = 6)		36		12					

A Case No.

B Sex

C Genotype

D Age when diagnosis was confirmed clinically

^a^ When starting as a blood donor

E Age when diagnosis was confirmed genetically

^a^ When starting as a blood donor

F Transferrin saturation before treatment

G Serum ferritin (µg/L) before treatment

(Normal range: females 10–220 µg/L, males 25–400 µg/L)

H Comment

^b^ Already blood donor when diagnosis was confirmed

I Age at onset of joint symptoms

J Initial joint location

^c^ MCP: metacarpophalangeal joints

K Delay in diagnosis (years)

L Total hip replacement (age)

M Total knee replacement (age)

N Total ankle replacement (age9

O Number of joints replaced

^d^ HTO: High tibial osteotomy

P No. of joints planned to be replaced

4 patients (cases 1–4) had been referred to me from other hospitals due to painful osteoarthrosis of one or both ankles. In 3 of these cases, the diagnosis had been made before referral by laboratory testing and liver biopsy but the genotype was determined at referral.

In patient 3, I suspected and confirmed the diagnosis. She proved to have a compound heterozygoty (C282Y/H63D). The patient had donated blood regularly, which explains her only modestly increased laboratory values. 3 other patients with HH (cases 5–7) who had been operated on for a total ankle prosthesis elsewhere were identified via the Swedish National Board of Health and Welfare. The correct diagnosis had been reported to the Swedish Ankle Arthroplasty Register (Henricson et al. 2007) in only one of these 3 cases.

None of the patients had reported any major ankle trauma. Preoperative radiographs of all ankles that had been replaced, and the as yet unreplaced ankles in cases 5 and 6, were scrutinized. The radiographic pattern was uniform, with reduction of the joint space, bony eburnation, cysts in the distal tibia and/or talus, and osteophytes. The latter were always located anteriorly at the neck of the talus and distal tibia, but sometimes also posteriorly. With few exceptions, the reduction of the joint space was located laterally and always anteriorly.

**Figure:**
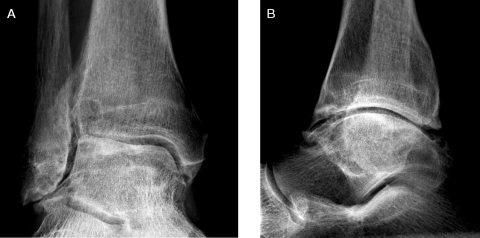
Case no 2. The right ankle preoperatively. This man had his right ankle and left knee replaced during the same anesthesia, at the age of 60. The following year his left ankle and right hip were replaced, also during the same anaesthesia. Pain and swelling of the knee started at the age of 40, but 13 years later it was first confirmed that he suffered from hemochromatosis. The ankles became symptomatic at the age of 42. Reduction of the joint space is seen anteriorly and laterally and there are osteophytes, notably anteriorly at the neck of the talus and distal tibia. Bony eburnation and cysts in the talus and distal tibia can also be seen.

## Discussion

Joint complaints are one of the most frequent symptoms of HH and they are often the first clinical manifestation of disease (McDonell et al. 1999, Inês et al. 2001, von Kempis 2001, Rihl and Kellner 2004). Although cirrhosis of the liver and cancer are important for mortality in patients with HH, arthropathy has the greatest effect on quality of life (von Kempis 2001).

The mechanism behind the arthropathy in HH is unknown. In HH, the sensitivity to cartilage damage is presumably increased and/or reparative capacity is reduced (Huch et al 1997). In most studies, no correlation has been found between serum ferritin levels and arthropathy. In contrast to other manifestations of the disease, the joint symptoms do not diminish after phlebotomy, or if so, only to a minor degree (Adams et al 1997, Sinigaglia et al 1997, McDonell et al. 1999, Rihl and Kellner 2004). Valenti et al. (2008) have, however, reported that 32 of 88 patients with phenotypically expressed HH had radiographically verified changes in the metacarpophalangeal (MCP) joints, and that the severity of these changes was influenced by the degree of iron overload.

In textbooks and most papers on HH, symptoms from—and radiographic changes in—the MCP joints are repeatedly described as typical for the disease, whereas little or no attention has been drawn to other joints. The reason for this is presumably that degenerative changes of other joints are much more common and cannot be distinguished from those that occur in HH.

Whether or not major joints (i.e. the hip, knee, ankle, shoulder, and elbow) are affected more often in HH than in the general population is unclear. Lunn et al. (2005) found 2 out of 116 patients who had undergone primary hip replacement to be C282Y homozygotes—a figure that is similar to the prevalence in the general population of Ireland. Curiously enough, substantially more patients—10 of 101 patients—who underwent revision of their hip were C282Y homozygotes. In a large population study, Adams et al. (2005) only found self-reported arthritis to be more common in males who were homozygous for H63D than in participants without HFE mutations. Walker et al. (2006) demonstrated an association between genotype and interleukin 1 receptor antagonist (IL1RN) levels in patients with HH and joint pain. Axford et al. (1991) described an increased incidence of hip involvement, and Rollot et al. (2005) suggested that osteonecrosis of the femoral head may be one expression of HH.

My observations and those of a few others (Bayley and Gardner 1998, Jacki et al. 1999, Schmid et al. 2003, Davies and Saxby 2006) indicate that the ankle may also be a key joint in HH. In the literature, I have found 5 papers that together reported on 13 patients who had had one ankle replaced and 3 papers that reported on HH patients who had had their hip joint replaced (Table 2). Here I report on another 7 patients who had 10 ankles and 6 other major joints replaced.

**Table 2.** HH and joint replacement as reported in the literature

First author	Year of publication	No. of patients	No. of hips	Thereof with AVN	No. of knees	No. of ankles	Total no. of joints	Comments
Jarde et al.	1977	1	–	–	–	1	≥ 1	A series of failed ankle prostheses
Montgomery et al.	1988	15	19	7	–	–	19	Case series
Hinterman	1999	2	–	–	–	2	–	A medium-term analysis of ankle prostheses
McDonell et al.	1999	109	–	–	–	–	>109	A survey of 2,851 HH patients
Lecoules et al.	2002	3	3				3	Case series
Davies and Saxby	2006	4	–	–	–	4	–	Case series
Fevang et al.	2007	4	–	–	–	4	–	Report from national register
Hosman et al.	2007	2	–	–	–	2	–	Report from national register
Own cases		7	4	?	2	10	16	Case series

– : not stated; AVN: avascular necrosis.

For unknown reasons, the ankle is less susceptible to osteoarthritis than the knee and hip, and symptomatic osteoarthritis of the ankle is uncommon even at advanced age.

All the patients in my study had one or two ankles replaced, and all but one patient also had at least one other joint that was replaced or diseased. Recently, Carroll (2006) reported a strong and statistically significant association between HFE gene mutations and primary OA in the ankle joint. Due to the frequent presence of OA of the second and third MCP joint in these patients, the same author also suggested the existence of a type-2 polyarticular OA phenotype that closely resembles the arthropathy of HH, which appears to be clinically differentiable from a type-1 OA or nodal generalized OA (NGOA).

There do not seem to be any radiographic phenomena that are typical of HH except for the reduction of joint space, bony eburnation, and broadening of the metacarpal heads seen in the MCP joints. This differs from what is usually seen in other types of arthritis.

The MRI appearance of arthropathy in HH cannot be distinguished from that of other types of degenerative osteoarthritis; the method cannot demonstrate the presence of iron in synovium, synovial fluid, or cartilage. It may be that the iron is below the threshold of detection by existing MRI techniques (Jager et al. 1997, Papakonstantinou et al. 2005). At surgery, the 7 ankles replaced by myself (cases 1–4) had the same appearance as in ordinary, degenerative osteoarthritis. In cases 2 and 4, the resected joint parts were examined histologically. No traces of iron were found.

The presence of long-standing joint pain and/or osteoarthritis in a person below the age of 55–60 years should thus arouse suspicion of HH if the symptoms cannot be related to another specific disease, e.g. seropositive rheumatoid arthritis, psoriasis, or arthritis urica. If more than one major joint is involved—notably bilateral ankle arthropathy without previous trauma—the suspicion is strengthened (Davies and Saxby 2006).

In such cases, plasma or serum iron levels, total iron-binding capacity (TIBC), and serum ferritin should be analyzed. An iron-saturation level (Fe/TIBC × 100) above 50% or an increased ferritin value should be followed by genetic testing. If this confirms that the patient is homozygous, or has a so-called compound heterozygoty for HH, the individual should be referred to a gastroenterologist for further examination. Joint symptoms do not appear to be influenced by early diagnosis and treatment, but awareness of the condition and positive screening may prevent patients and their relatives from undergoing the more serious consequences of HH by regular phlebotomy.

## References

[CIT0001] Adams PC, Deugnier Y, Moirand R, Brissot P (1997). The relationship between iron overload, clinical symptoms, and age in 410 patients with genetic hemochromatosis.. Hepatology.

[CIT0002] Adams PC, Reboussin DM, Barton JC, McLaren CE, Eckfeldt JH, McLaren GD, Dawkins FW, Acton RT, Harris EL, Gordeuk VR, Leiendecker-Foster C, Speechley M, Snively BM, Holup JL, Thomson E, Sholinsky P (2005). Hemochromatosis and iron-overload screening in a racially diverse population.. N Engl J Med.

[CIT0003] Axford JS, Bomford A, Revell P, Watt I, Williams R, Hamilton EB (1991). Hip arthropathy in genetic hemochromatosis. Radiographic and histologic features.. Arthritis Rheum.

[CIT0004] Bailey EJ, Gardner AB (1998). Hemochromatosis of the foot and ankle. Report of three cases and review of the literature.. Clin Orthop.

[CIT0005] Carroll GJ (2006). Primary osteoarthritis in the ankle joint is associated with finger metacarpophalangeal osteoarthritis and the H63D mutation in the HFE gene: evidence for a hemochromatosis-like polyarticular osteoarthritis phenotype.. J Clin Rheumatol.

[CIT0006] Davies MB, Saxby T (2006). Ankle arthropathy of haemochromatosis: a case series and review of the literature.. Foot Ankle Int.

[CIT0007] Elmberg M, Hultcrantz R, Ekbom A, Brandt L, Olsson S, Olsson R, Lindgren S, Lööf L, StÅl P, Wallerstedt S, Almer S, Sandberg-Gertzén H, Askling J (2003). Cancer risk in patients with hereditary hemochromatosis and in their first-degree relatives.. Gastroenterology.

[CIT0008] Feder JN, Gnirke A, Thomas W, Tsuchihashi Z, Ruddy DA, Basava A (1996). A novel MHC class I-like gene is mutated in patients with hereditary haemochromatosis.. Nat Gen.

[CIT0009] Fevang B-T, Lie S, Havelin L, Brun J, Skredderstuen A, Furnes O (2007). 257 ankle arthroplasties performed in Norway between 1994 and 2005.. Acta Orthop.

[CIT0010] Henricson A, Skoog S, Carlsson Å (2007). The Swedish ankle arthroplasty register. An analysis of 531 arthroplasties between 1993 and 2005.. Acta Orthop.

[CIT0011] Hinterman B (1999). Short- and mid-term results with the STAR total ankle prosthesis.. Orthopäde.

[CIT0012] Hosman AH, Mason RB, Hobbs T, Rothwell AG (2007). A New Zealand national joint registry review of 202 total ankle replacements followed for up to 6 years.. Acta Orthop.

[CIT0013] Huch K, Kuettner KE, Dieppe P (1997). Osteoarthritis on the ankle and knee joints.. Semin Arthritis Rheum.

[CIT0014] Inês LS, da Silva JA, Malcata AB, Porto AL (2001). Arthropathy of genetic hemochromatosis: a major and distinctive manifestation of the disease.. Clin Exp Rheumatol.

[CIT0015] Jacki SH, Uhl M, Adler CP, Peter HH, von Kempis J (1999). Predominant ankle arthropathy in hereditary haemochromatosis.. Rheumatology Oxford.

[CIT0016] Jager HJ, Mehring U-M, Gotz GF, Neise M, Erlemann R, Kapp HJ, Mathias KD (1997). Radiological features of the visceral and skeletal involvement of hemochromatosis.. Eur Radiol.

[CIT0017] Jarde O, Gabrion A, Meire P, Trinquir-Lautard JL, Vives P (1997). Complication and failures of total ankle prosthesis. Apropos of 21 cases.. Rev Chir Orthop Reparatrice Appar Mot.

[CIT0018] Lecoules S, el Maghraoui A, Damiano J, Lechevalier D, Magnin J, Eulry F (2002). Hip arthroplasty in genetic hemochromatosis. Report of 5 cases.. Rev Med Interne.

[CIT0019] Lunn JV, Gallagher PM, Hegarty S, Kaliszer M, Crowe J, Murray P, Bouchier-Hayes D (2005). The role of hereditary hemochromatosis in aseptic loosening following primary total hip arthroplasty.. Orthop Res.

[CIT0020] McCune CA, Ravine D, Carter K, Jackson HA, Hutton D, Hedderich J, Krawczak M, Worwood M (2006). Iron loading and morbidity among relatives of HFE C282Y homozygotes identified either by population genetic testing or presenting as patients.. Gut.

[CIT0021] McDonell S, Preston B, Jewell S, Barton J, Edwards C, Adams PC, Yip R (1999). A survey of 2,851 patients with haemochromatosis: Symptoms and response to treatment.. Am J Med.

[CIT0022] Montgomery KD, Williams JR, Sculco TP, DiCarlo E (1998). Clinical and pathologic findings in hemochromatosis hip arthropathy.. Clin Orthop.

[CIT0023] Papakonstantinou O, Mohana-Borges AVR, Campell L, Haghighi P, Resnick D (2005). Hip arthropathy in a patient with primary hemochromatosis: MR imaging findings with pathological correlation.. Skeletal Radiology.

[CIT0024] Rihl M, Kellner K (2004). Die Artopathie der Heriditären Hämochromatose.. Z Rheumatol.

[CIT0025] Rollot F, Wechsler B, du Boutin le TH, Degennes C, Amoura Z, Hachulla E, Piette JC (2005). Hemochromatosis and femoral head aseptic osteonecrosis: a nonfortuitous association?. J Rheumatol.

[CIT0026] Schmid H, Struppler C, Braun GS, Kellner W, Kellner H (2003). Ankle and hindfoot arthropathy in hereditary hemochromatosis.. J Rheumatol.

[CIT0027] Sinigaglia L, Fargion S, Fracanzani AL, Binelli L, Battafarano N, Varenna M, Piperno A, Fiorelli G (1997). Bone and joint involvement in genetic hemochromatosis: role of cirrhosis and iron overload.. J Rheumatol.

[CIT0028] Valenti L, Rametta R, Dongiovanni P, Maggioni M, Ludovica Fracanzani A, Zappa M, Lattuada E, Roviaro G, Fargion S (2008). The hand arthropathy of hereditary hemochromatosis is strongly associated with iron overload.. J Rheumatol.

[CIT0029] von Kempis J (2001). Arthropathy in hereditary hemochromatosis.. Curr Opin Rheumatol.

[CIT0030] Walker EJ, Riddell J, Rodgers HJ, Bassett ML, Wilson SR, Cavanaugh JA (2006). IL1RN genotype as a risk factor for joint pain in hereditary haemochromatosis.. Ann Rheum Dis.

